# COMSKIL: a communication skills training program for medical students

**DOI:** 10.3205/zma001479

**Published:** 2021-04-15

**Authors:** Claudia Gebhardt, Anja Mehnert-Theuerkauf, Tim Hartung, Anja Zimmermann, Heide Glaesmer, Heide Götze

**Affiliations:** 1 University Medical Center Leipzig, Department of Medical Psychology and Medical Sociology, Leipzig, Germany; 2 University Medical Center Leipzig, LernKlinik Leipzig, Leipzig, Germany

**Keywords:** teaching concept, medical school education, patient-physician communication, communication skills, COMSKIL

## Abstract

**Objectives: **Training communication skills has come to be recognized as a vital aspect of medical school education. A medical communication course based on the COMSKIL Communication Skills Training (CST) Program was developed, integrated into the core curriculum, and evaluated at the Leipzig University Medical School.

**Methods: **Between October 2016 and July 2017, 312 medical students (mean age 21.80 years; 62% male) participated in the medical communication course. Each course unit was evaluated via questionnaires specifically designed to address the theoretical and practical content of the curriculum. The items correspond to the material covered in each course unit. Students responded using a 5-point-Likert scale (1=“not at all helpful”, 5=“extremely helpful”) to rate the degree to which the course helped them learn about the subject matter and train the skills covered in the curriculum.

**Results: **The average score for the first part of the course (theoretical foundations) was *M*=3.69 (*SD*=0.35). The second part received a similar rating (*M*=3.84; *SD*=0.73). The role play exercises with actor-patients received a score of *M*=4.27 (*SD*=0.62). In an overall evaluation at the end of the course, students rated the administration of the course (setting, etc), knowledge gained, and skills trained with a score of *M*=4.11 (*SD*=0.66). The role play exercises received an overall score of *M*=4.36 (*SD*=0.61).

**Conclusion:** A new curriculum for teaching medical students patient-physician communication skills based on the COMSKIL CST program was established at the University of Leipzig. The goal of this course is to teach students about the kinds of communication scenarios they will encounter in their future working lives as care providers and equip them with the fundamental communication techniques and skills they need to successfully handle those situations. A formal evaluation of the program resulted in satisfactory findings, indicating that it is well suited for use in medical universities.

## Zusammenfassung

**Hintergrund: **Der Erwerb und die Förderung von Kommunikationsfertigkeiten für die Gesprächsführung sind wesentliche Ziele der ärztlichen Aus- und Weiterbildung. An der Medizinischen Fakultät der Universität Leipzig wurde ein Lehrkonzept für die Ausbildung in ärztlicher Gesprächsführung entwickelt und etabliert, das auf dem COMSKIL-Programm beruht. In der vorliegenden Arbeit werden das Lehrkonzept sowie die Evaluation durch die Studierenden vorgestellt.

**Methodik:** Von Oktober 2016 bis Juli 2017 haben 312 Studierende (mittleres Alter 21,80 Jahre; 62% männlich) den COMSKIL-Kurs zur ärztlichen Gesprächsführung absolviert. Die Evaluation erfolgte anhand von Fragebögen, die spezifisch auf die theoretischen und praktischen Kursinhalte zugeschnitten waren. Studierende sollten mittels 5-Punkt-Likert Skalen („gar nicht hilfreich“ bis „sehr hilfreich“) angeben, inwiefern ihnen der Gesprächsführungskurs dabei geholfen hat, Kenntnisse und Fertigkeiten zu den vorgestellten Inhalten bzw. durch die durchgeführten Rollenspiele zu erwerben.

**Ergebnisse: **Die mittlere Bewertung der Inhalte im ersten Kursabschnitt lag bei *M*=3,69 (*SD*=0,35). Im zweiten Kursabschnitt war die Bewertung ähnlich (*M*=3,84; *SD*=0,73). Für die Rollenspiele mit Simulationspatientinnen und -patienten lag die Bewertung bei *M*=4,27 (*SD*=0,62). Eine globale Evaluation des Kommunikationskurses zum letzten Seminartermin zeigte hinsichtlich der Rahmenbedingungen und der Vermittlung von Wissen, Fähigkeiten und Fertigkeiten eine gute Bewertung von *M*=4,11 (*SD*=0,66). Die Rollenspiele wurden insgesamt mit *M*=4,36 (*SD*=0,61) bewertet.

**Fazit: **Die Inhalte und didaktischen Methoden des auf dem COMSKIL Konzepts basierenden Programms zielen darauf ab, Studierende für verschiedene Gesprächssituationen zu sensibilisieren und ihnen grundlegende Kommunikationstechniken und -fertigkeiten zu vermitteln. Die Evaluation zeigte zufriedenstellende Ergebnisse und spricht für die gute Anwendbarkeit des Programms in der universitären Lehre der Humanmedizin.

## Introduction

Communication plays a central role in the everyday work of being a doctor. In the “Masterplan Medizinstudium 2020“ (Medical Studies Master Plan 2020) [1] enacted in 2017 by the German Federal Ministry of Health and Federal Ministry of Education and Research (BMG) together with all of the German state-level ministries of health and cultural affairs, special emphasis is placed on the importance of medical communication skills due to the significant impact they have on the quality of patient-physician relationships, treatment success rates, and patient wellbeing. Over time, it is has come to be accepted as a matter of fact that care providers' communication skills impact important healthcare outcome parameters. For patients, these include, among others: increased satisfaction, comprehension, treatment success, and treatment adherence as well as reduced burden. At the same time, doctors benefit from reduced rates of depression and burnout and improved wellbeing and job satisfaction [2], [3], [4], [5], [6], [7]. The Master Plan 2020 therefore requires that medical schools teach students the fundamentals of good patient-physician communication as a core subject in order to adequately prepare them for the daily demands of their future profession. Furthermore, it emphasizes that the communication skills in question can be demonstrably improved when taught as early as possible and consistently trained thereafter [8], [9]. 

The German National Competence-based Learning Objectives Catalogue for Undergraduate Medical Education (NKLM; [http://www.nklm.de]) provides practical guidance by specifying in detail the subjects and skills that should be taught. The learning objectives related to communication are formulated in chapter 14c, “Patient-Physician Communication”. Quality standards for teaching patient-physician communication skills are also outlined in the longitudinal “Mustercurriculum Kommunikation in der Medizin” (LongKomm [10]) [prototype curriculum for medical communication].

The implementation of these aims and standards for teaching communication skills in medical schools has generated a wide range of didactic concepts, of which however only a few have been evaluated and published [11], [12], [13], [14], [15], [16]. Beyond these, exists a series of evaluated CST programs (COMSKIL [17], Oncotalk [18], Kompass [19] COM-ON-p [20]) that have been developed to address aspects of communication between healthcare providers and patients but are not tailored to medical university teaching requirements. The aim of the project presented here was therefore to modify, evaluate, and publish an established communication skills training program adapted specifically for use in the medical school setting.

## Project description

A multi-semester longitudinal “Communication” curriculum was integrated into the required coursework offered at the University of Leipzig beginning in the Winter Semester of 2016/2017. The course is comprised of an interdisciplinary synthesis of communication theory and clinical practice. Students begin taking the course during their pre-clinical studies and continue over the entire length of their time in medical school. An actor-patient program was developed for this purpose. Approval for carrying out an evaluation of the course was granted by the ethics board of the University of Leipzig (149/17 – ek). The first part of the longitudinal communication curriculum is a course on medical communication offered in the third and fourth semester of students' pre-clinical education. One key aim of this endeavor was to determine how successfully a CST program which was already well established and evaluated could be adapted to the requirements of the medical school setting. The COMSKIL [17] communication skills training program, introduced below, was consequently chosen for this purpose. 

### The COMSKIL communication skills training program

The COMSKIL program was developed by Kissane et al. at the Memorial-Sloan-Kettering Cancer Center (MSKCC) [17], [21], [22]. Though originally designed for use in oncology, it is transferable to other medical areas as well. It is an interdisciplinary program for doctors, nurses, and other healthcare workers charged with caring for patents. The training program is based on the following theoretical models:

1. the goals-plans-action theory [23];
2. sociolinguistics theory [24];
3. and the lay-etiological “common sense model of illness” as it relates to illness-related self-regulation [25].

According to “goals-plans-action” theory [24], it is essential to first establish communication goals relevant to the situation at hand, next devise corresponding communication plans, and finally to enact those plans with concrete communication behavior. The sociolinguistic approach [24], especially as it pertains to person-centered communication, is based on the idea that conversational goals can be achieved by utilizing a variety of techniques. This however is based on the presumption that a person’s capacity for perceiving and being considerate of their conversation partner's needs is sufficient enough to enable them to choose the appropriate technique. According to the common-sense-model [25], patients have their own conceptual model of their illness and treatment effects and successful medical communication relies on care providers simultaneously seeking to understand that person's model and respectfully working to amend it where necessary. This patient-centered approach seeks to ensure that all of the conversation partners involved reach a shared understanding of the illness and treatment possibilities, a cornerstone of shared decision making [26] that can directly impact treatment outcomes. 

**Figure 1:**
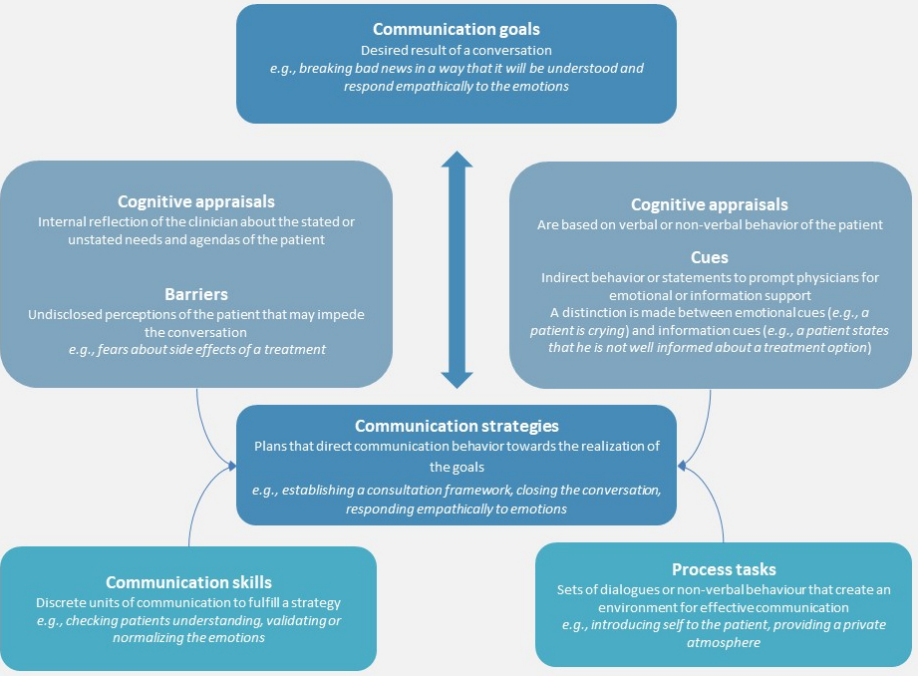
COMSKIL model (adapted from Kissane et al., 2012)

The COMSKIL model synthesizes these theories and explicitly defines the central components of a medical consultation using a model comprised of the following elements: communication goals, communication strategies, communication techniques, process tasks, and cognitive assessments (see figure 1 (Fig. 1)). This has the advantage of clearly identifying which skills are essential for communication and therefore need to be taught both in medical school and continued education programs [17]. According to this model, the ability to set communication goals is key. These in turn determine which communication strategies are chosen and implemented. The concrete execution is realized via specific communication techniques (i.e. questioning techniques, verbalization, normalization) and the observance of process assignments (i.e. choosing an appropriate setting for the conversation). In pursuing communication goals, it is also important to carry out cognitive assessments (i.e. comprehending when a patient needs more information or emotional support). The goal of the communication skills training program is to teach a range of communication skills and techniques that care providers can flexibly apply in response to a patient's needs and the demands of the situation at hand. An example of this is the “cognitive assessments” unit that teaches and trains various techniques (i.e. normalization, verbalization) that equip care providers for dealing with their patients' emotions. 

The original English-language version of the COMSKIL program is comprised of ten modules:

1. sharing serious news,
2. discussing prognoses,
3. interactions with patients and families,
4. responding to emotions like frustration and anger,
5. communicating via interpreters,
6. shared decision making and explaining clinical studies,
7. dealing with defensiveness and avoidance,
8. communication with long-term survivors,
9. handling disease recurrence,
10. handling issues related to end-of-life care, death, and dying.

Theoretical foundations and practical examples are provided for each of these specific situations in every module of the program. In the concept's original design, the communication skills training takes place with small groups (max. 6 people) over the course of a two-day workshop. Every group has access to two trainers, one of whom is a medical specialist, and the other who is grounded in a psychosocial discipline. The training sessions primarily revolve around role playing exercises conducted with simulation patients (SP). Every module has an accompanying brochure outlining its theoretical content and practical examples. A German-language version of the program revised for relevance to the German healthcare system exists as well [27], [28]. As COMSKIL is a well-evaluated teaching concept dealing with a range of subjects also outlined in the German National Competence-based Learning Objectives Catalogue for Undergraduate Medical Education [http://www.nklm.de], it was deemed a suitable program to be adapted and evaluated for use in medical school setting. 

#### Re-conception of the COMSKIL communication skills training (CST) program for use in medical schools 

**Figure 2:**
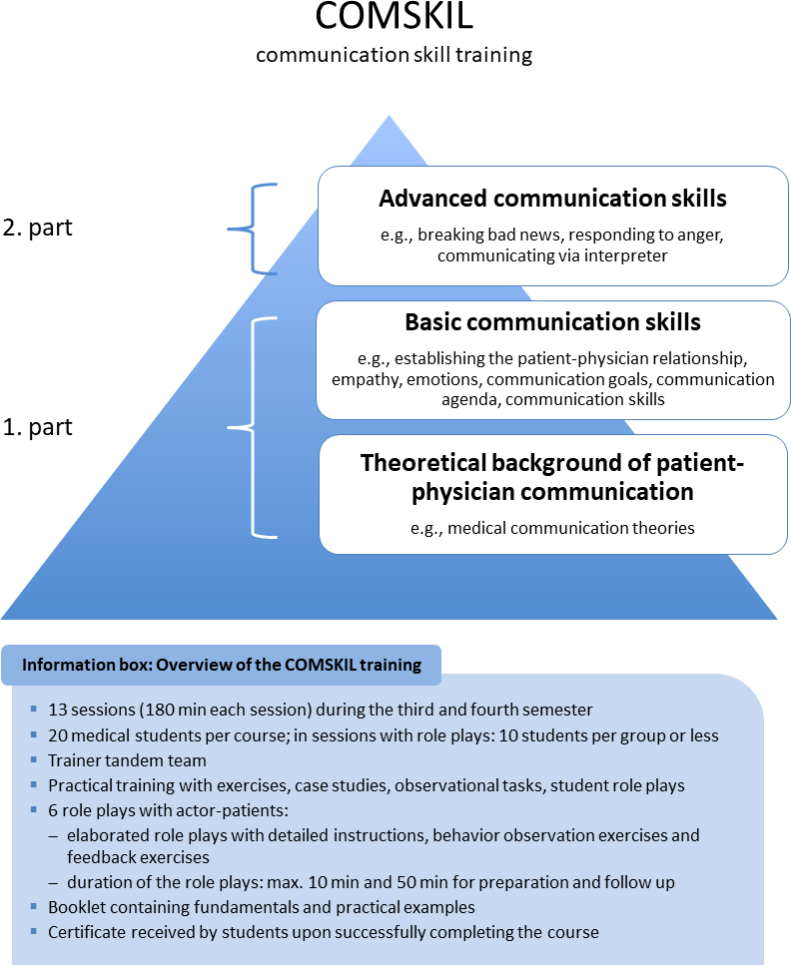
Overview of the COMSKIL communication skill training program for the use in medical schools

The course on patient-physician communication is integrated into the framework of the university’s obligatory pre-clinical curriculum. All medical students are required to take the course during their third and fourth semesters. The course encompasses two credit hours but is offered in blocks of four credit hours each. Each course group consists of approximately twenty students. As outlined in the German National Competence-based Learning Objectives Catalogue for Undergraduate Medical Education paragraph on medical communication and in the Längschnittcurriculum Medizin (Longitudunal Curriculum for Medicine) [10] [http://www.nklm.de], the general theories and models pertaining to patient-physician communication as well as fundamental communication skills are an essential element of medical education. Because the original COMSKIL program is based on processing various “real-life” communication scenarios but the participating medical students are still in the pre-clinical part of their training and therefore rarely in contact with patients, the decision was made to structure the course in a modular way. Thus the material presented gradually progresses from covering general fundamentals to focusing on specific medical communication scenarios, including training exercises conducted with actor-patients (simulation patients: SP) (see figure 2 (Fig. 2)). 

The first part of the course (third semester) introduces the COMSKIL CST model and other medical communication theories as well presenting a range of communication techniques (i.e. starting a conversation, techniques for posing a question, active listening, the role of observing and assessing, the meaning of emotions and cognition). The content of each of the sessions is presented in table 1 (Tab. 1). The second part of the course (fourth semester) builds on this by applying that knowledge to specific scenarios outlined in the COMSKIL training concept (i.e. Breaking bad news, Discussing prognoses, Interacting with families). The introduction of the specific subjects is timed to coordinate with students’ general coursework to ensure that they are grounded in the medical knowledge needed for the scenario at hand. Students are also prepared for the role playing exercises with instructions sketching out the background of the role, the type of conversation that may come up, and the tasks to be accomplished in the interactions. They are further provided with a list of references they can use to brush up their knowledge about the medical subjects they will encounter in the role play. An overview of the specific content of each session and role play is presented in attachment 1 . 

The course is taught using a variety of didactic methods, materials, media, and practical exercises to enable a multi-faceted learning process including theoretical, reflective, experiential, and interactive work (see table 2 (Tab. 2)). Existing evidence has shown that the role playing exercises, giving and receiving feedback, and discussion of practical examples or implications of theoretical frameworks are particularly effective [29]. 

As is true of the original COMSKIL training concept, the courses are led by tandem faculty teams, of whom both members are active in the field of psycho-sociology (one with an emphasis on scientific teaching and the other a practicing clinician). All of the instructors received extensive training beforehand. For the sessions featuring role plays with SP, “peer teaching” is practiced by pairing each faculty team with a teaching assistant, in this case, upper-level medical students who have previously completed the course. The SP are (lay) actors of various ages who have received intensive training enabling them to credibly embody the roles and give qualified feedback afterwards. In order to optimize the experience of the role plays and debriefing sessions with SP, the course group is divided into smaller groups of ten students or less. 

## Evaluation

The evaluation of the COMSKIL medical communication course was carried out from October 2016 through July 2017. All of the students enrolled in the medical communication course were asked to complete a written survey at the end of each session. For purposes of data protection, the survey was conducted on a purely voluntary and anonymous basis. The only personal information collected was the age and gender of the students, and this information was handled such that it could not be used to identify individual participants. By asking the students to record their age but not their birthday, the level of identity protection was improved by decreasing the degree of detail captured in the data. Once the data had been recorded, the questionnaires were stored in a centralized archive. It was not necessary to apply for ethical approval for this particular evaluation because it fell within the domain of standard quality control surveys conducted on a regular basis at the medical school. 

The survey questions were developed specifically to evaluate the course presented here and are based on the content of the individual course sessions. During the first part of the course, students were asked how helpful the course had been for gaining new understanding and skills relevant to the topics covered (i.e. *“How helpful was the course for learning about and training questioning techniques?”*). The content of the items is presented in table 1 (Tab. 1). The responses were given using a 5-point Likert scale (1=*not at all*, 2=*a little*, 3=*fairly*, 4=*greatly*, 5=*very greatly*). During the second part of the course, both the theoretical components and the role plays were evaluated. The response format was slightly altered. The students responded using a 5-point Likert scale (1=*strongly disagree*, 2=*somewhat disagree*, 3=*neither agree nor disagree* 4=*agree* 5=*strongly agree*) to score the extent to which the module had aided them in improving their knowledge and skills relevant to the subjects covered (i.e.* “The skills I learned in this module will help me deal more empathetically with difficult patients”*.) and whether they were satisfied with the role plays (i.e. *“The role play was well-prepared.”*, *“The level of difficulty of the role play was appropriate.”*, *“The role play made it possible to practically apply the theoretical communication basics of interacting with patients.”*). The content of the items is presented in attachment 1 . While the questions concerning the theoretical material vary, the questions about the role plays remain the same for every session. 

After the last session of the second part of the course, a final survey was conducted to rate the entire two-semester course using ten items (see table 3 (Tab. 3)). The students were asked to share their impressions concerning how the course was run, the teaching of theoretical concepts and practical skills, the difficulty of the course, and perceived improvements in their communication skills. They were also asked to rate the content and administration of the role plays, i.e. themes chosen, feasibility, difficulty, and how useful they had been for solidifying the theoretical fundamentals covered in the course. Here too the responses were given using a 5-point Likert scale (1=*strongly disagree*, 2=*somewhat disagree*, 3=*neither agree nor disagree*, 4=*agree*, 5=*strongly agree*). 

The analysis of the questionnaires was conducted descriptively with SPSS [30]; Averages and standard deviations were calculated for the single items for each session as well as total averages for the session in question and the role plays (see table 1 (Tab. 1), attachment 1 and table 3 (Tab. 3)).

## Results

### Sample

A total of 312 students (15 groups) completed the medical communication course during the evaluation period. The students were in their third semester of medical school when they took the first part of the course and in their fourth semester when they completed the second. They ranged in age from 18 to 47 years old (*M*=21.80, *SD*=.44). 62% of the participants were male. The participation rate ranged between 25% and 95%. 

#### Evaluation results

The average scores given the first part of the course (see table 1 (Tab. 1)) were between *M*=3.66 (*SD*=0.73) and *M*=3.81 (*SD*=0.78), meaning that, on average, the students found the course rather helpful for learning about the material that had been covered. 

The total average of the evaluation of the first part of the course was *M*=3.69 (*SD*=0.35). The scores for the theoretical content of the second part of the course (see attachment 1 ) were between *M*=3.62 (*SD*=0.89) and *M*=4.09 (*SD*=0.65). The overall score for the theoretical material was *M*=3.84 (*SD*=0.73). The role playing exercises received scores between *M*=4.14 (*SD*=0.70) and *M*=4.39 (*SD*=0.61), with an overall average of *M*=4.27 (*SD*=0.62). The global evaluation of the communication course conducted at the last session (see table 3 (Tab. 3)) resulted in an average score of *M*=4.11 (*SD*=0.66) for the administration of the course and the knowledge and skills taught. The role plays were given a score of *M*=4.36 (*SD*=0.61). Specifically, this means that the students found the level of difficulty of the role playing exercises appropriate and that they were satisfied with the choice of themes addressed and how the role playing sessions were run. Furthermore, the students agreed that the course had helped them improve their communication skills. 

## Further development of the medical communication course

Various modifications were made to the medical communication course based on student feedback, for example, more role playing exercises were added and some of the theoretical components were revised or shortened. In the original design, the first part of the course focused solely on the theoretical fundamentals and basic communication skills specified in the German National Competence-based Learning Objectives Catalogue [4], while role playing exercises with SP were not introduced until the second part. The students’ responses indicated however that they had found the role playing exercises very helpful and wished they had been introduced earlier. The current medical communication course curriculum therefore now includes two sessions in the first part of the course that feature role playing with SP; one on the subject “medical history consultation” was newly conceived, and the second, an exercise dealing with the theme “communicating with older patients”, was shifted from the second part of the course to the first. A newly designed role play with the subject “communication with patients using interpreters in a multi-person setting” was inserted in its place. As such, the modules in the first part of the course now include more practical work while nevertheless retaining their original emphasis on laying the theoretical groundwork and teaching the fundamentals skills of communication. 

## Discussion

Previous studies have shown that good patient-physician communication not only improves quality of care and the doctor-patient relationship [31], [32]; it positively impacts doctors’ health as well by reducing their levels of stress [33]. These results illustrate that high-quality medical communication training in medical schools can contribute to improving the overall quality of care our medical systems provide [34]. Over time, communication skills training has become an integral part of the education offered by German-language medical schools. Almost every degree program includes communication skills training featuring SP and feedback during students’ second and third years of study [34]. 

At the University of Leipzig, a comprehensive medical communication curriculum was developed and established based on the COMSKIL-training program [17]. The predominantly positive evaluation results show that the curriculum was well received by the students and demonstrate that an established CST program can successfully be adapted for use in the medical school setting. In particular, the high satisfaction ratings for the role playing exercises with simulation patients show that the students found this part of the course to be especially valuable. This outcome directly impacted the further development of the teaching concept, which was modified to include more practical exercises and work with SP. This is also conforms with the “Masterplan Medizinstudium 2020” [1] requirement that communication skills training be introduced as early as possible. Other studies have also proven that practical learning is more effective at producing positive results [28]. A further advantage of working with SPs is that the conversations simultaneously feel quite real and take place in a safe space. Creating this safe space was an important goal in the development of this course for third and fourth semester students as many of them had little to no previous medical communication experience. 

The integration of the COMSKIL-training program into the “longitudinal communication curriculum” our medical school offers has significantly contributed to improving the communication skills training our medical students receive. Results from studies done in the field of dentistry indicate that a longitudinal communication curriculum can contribute to both increasing positive and decreasing negative attitudes towards learning communication skills [35].

### Limitations

There are some limitations of the program’s evaluation that warrant mentioning. Because the results are based on subjective assessments, the feasibility of making statements about how much the participants’ communication skills actually improved remains limited. We recommend that future evaluations of the program include objective methods for measuring students’ progress, for example via external assessments or graded exams. The use of an unvalidated questionnaire presents a further limitation. It was however important for the purposes of this initial evaluation of the concept that each of the course modules be assessed individually. That being the case, a questionnaire had to be custom-designed to cover all of the topics addressed in the course and capture detailed information on students' ratings of its contents. The fluctuating number of participants who completed the questionnaires must also be taken into consideration. Because the evaluation was conducted on a voluntary basis and students were allowed one excused absence, the number of respondents varied considerably from one course group to the next. 

## Conclusions

By modifying the COMSKIL Communication Skills Training Program, it was possible to develop an educational concept for teaching medical communication in the university medical school setting that complies with the German National Competence-based Learning Objectives Catalogue for Undergraduate Medical Education (NKLM) [http://www.nklm.de] and encompasses the content necessary to fulfill the standard requirements of a longitudinal communication curriculum. The course's content and didactic methods were adopted with the aim of preparing students for various consultation scenarios they will encounter in their future work lives and to teach them basic skills that can be flexibly adapted to the demands of the situation at hand. The program was evaluated by the student participants, whose scores reflected satisfactory results. The next planned evaluation of this teaching concept will include measures for objectively assessing students' progress developing their communication skills. 

## Competing interests

The authors declare that they have no competing interests. 

## Supplementary Material

Content and evaluation of each of the sessions of the second part of the COMSKIL communication skills training course

## Figures and Tables

**Table 1:**
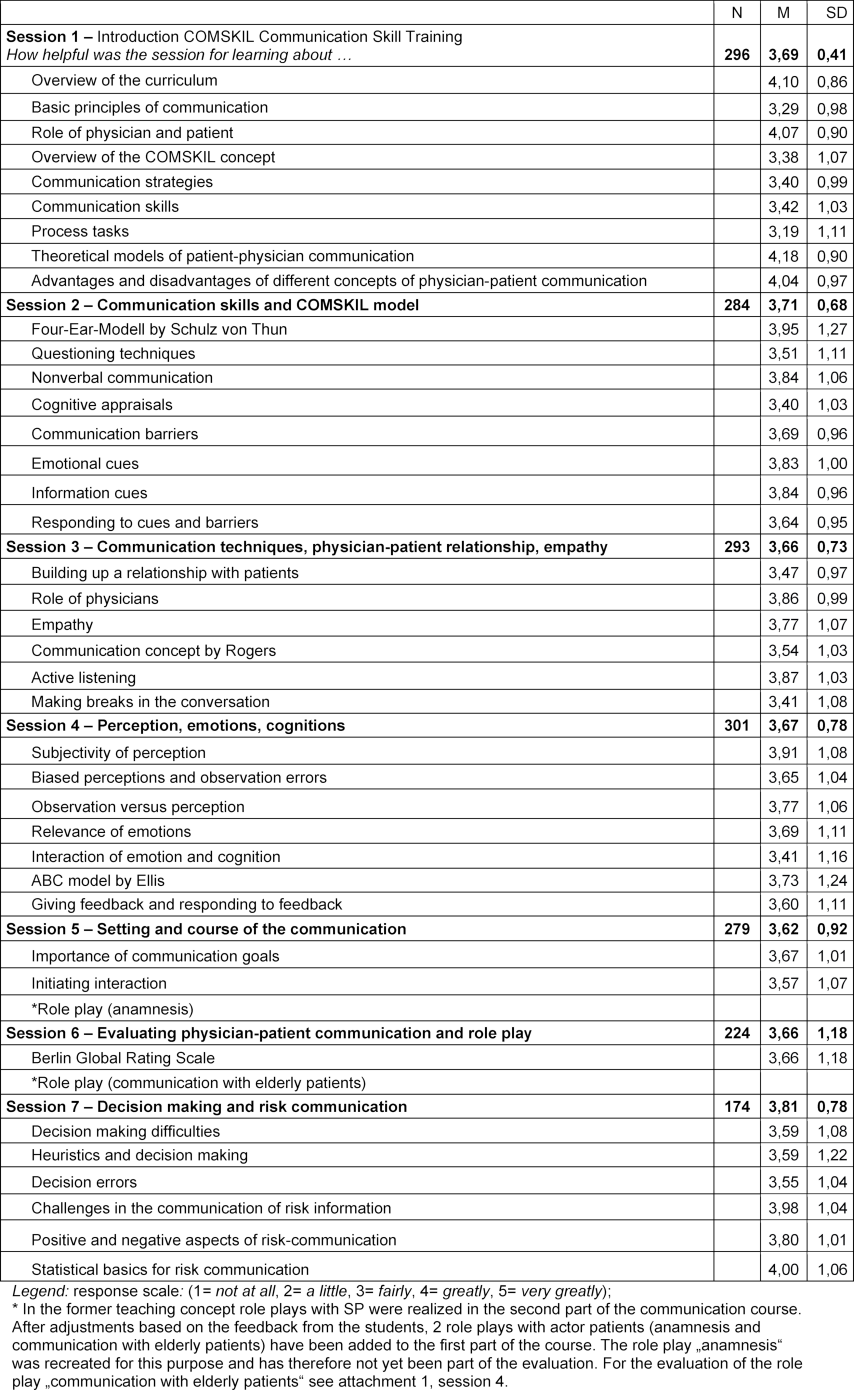
Content and evaluation of each of the sessions of the first part of the COMSKIL communication skills training course

**Table 2:**
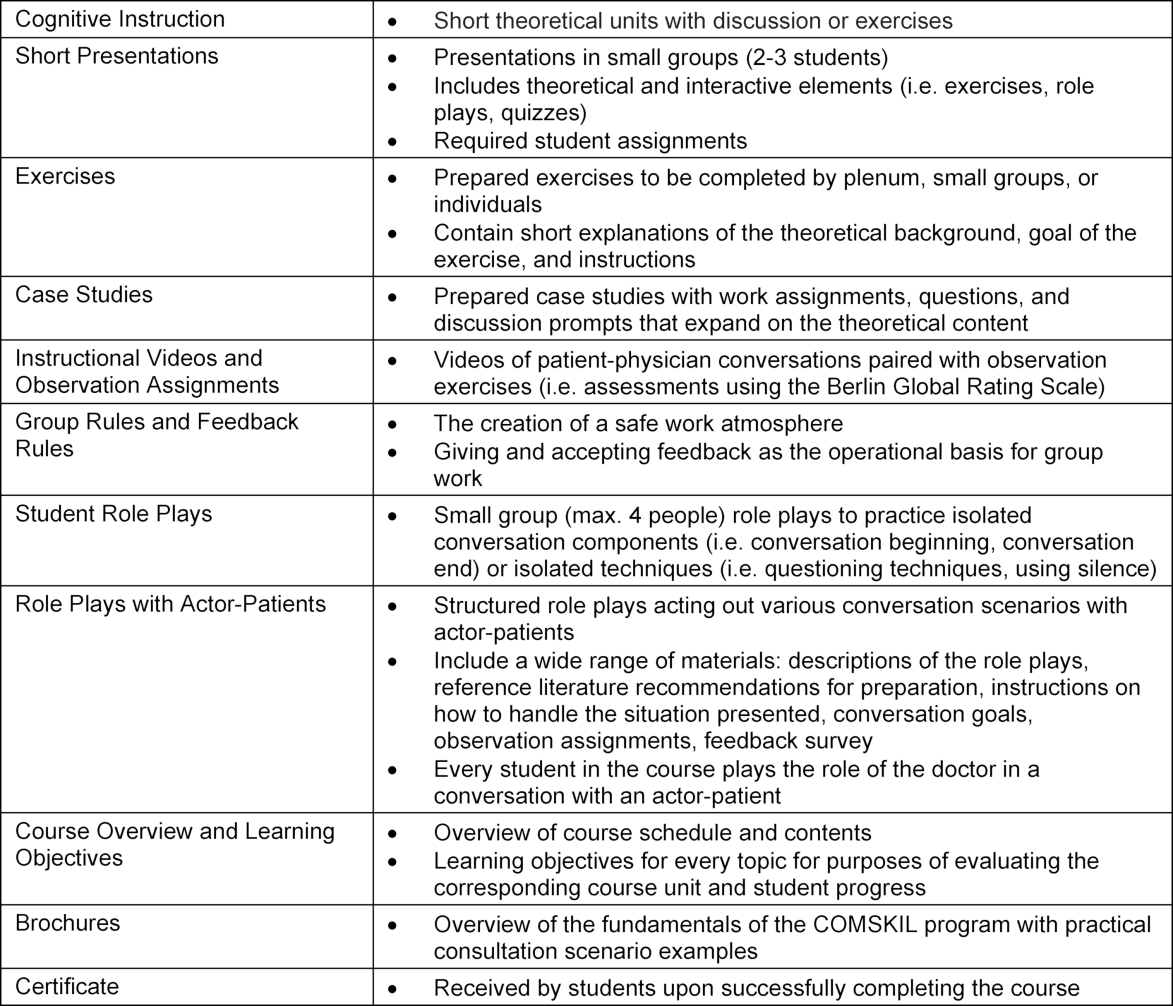
Overview of the didactic methods and materials used in the communication skills training course

**Table 3:**
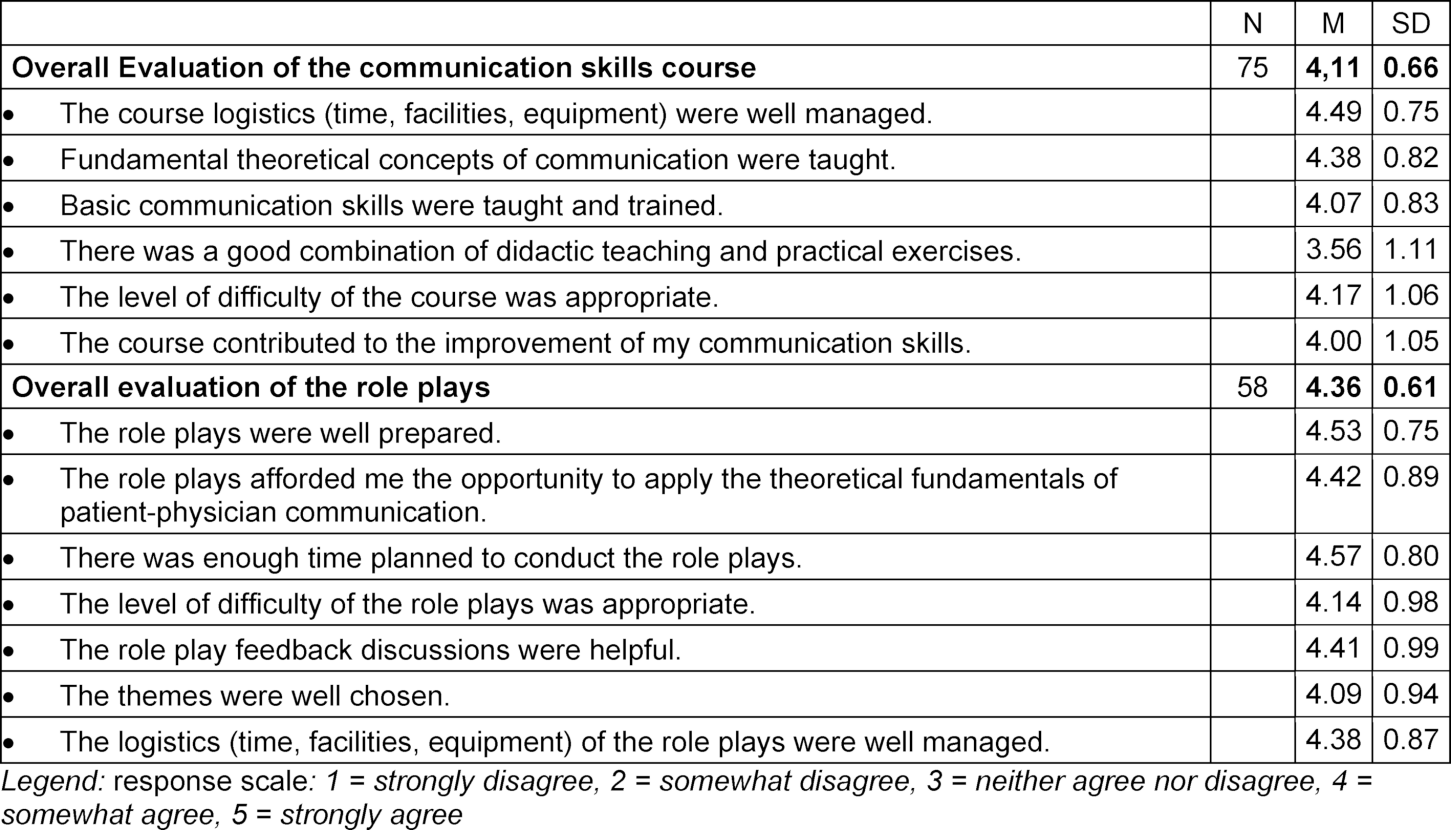
Total evaluation of the COMSKIL communication skills training course
