# Supplementary material for: COMSKIL: a communication skills training program for medical students
Source: GMS J Med Educ. 2021 Apr 15;38(4):Doc83. doi: 10.3205/zma001479 (PMC8136352; doi:10.3205/zma001479)
Supplement: Content and evaluation of each of the sessions of the second part of the COMSKIL communication skills training course [file JME-38-4-83-s-001.pdf]

Attachment 1: Content and evaluation of each of the sessions of the second part of the COMSKIL communication skills training course

	N	M	SD
**Session 1 – Breaking bad news**			
**Theoretical units**<br>*The theoretical units have aided in improving knowledge and skills relevant to …*	153	**4,09**	**0,65**
Definition of bad news		4,32	0,81
Communication challenges and difficulties in delivering bad news		4,09	0,80
Communication goals and strategies for providing information in an effective way		4,05	0,73
Skills and process tasks to convey threatening information in an appropriate way		3,83	0,90
**Session 2 – Responding to difficult emotions**			
**Theoretical units**	187	**3,86**	**0,67**
Empathic reactions to the patient's emotions		4,18	0,83
Empathically respond to angry patients		3,91	0,82
Goals and strategies for an appropriate communication with angry patients		3,87	0,80
Skills and process tasks for an effective communication with angry patients		3,46	1,00
**Role play (patient with tendonitis)**	174	**4,39**	**0,61**
The role play was well-prepared.		4,41	0,76
The role play made it possible to practically apply the theoretical communication basics of interacting with patients.		4,40	0,82
There was enough time planned to conduct the role play.		4,44	0,85
The level of difficulty of the role play was appropriate.		4,29	0,81
The role play feedback discussion was helpful.		4,40	0,82
**Session 3 – Run a family meeting**			
**Theoretical units**	231	**3,80**	**0,70**
Complexity of the communication with patients and their relatives		3,92	0,89
Challenges of involving and supporting relatives		3,98	0,82
Goals and core elements for the communication with patients and their relatives		3,84	0,87
Skills and process tasks for an adequate and effective communication with patients and their families		3,44	1,03
**Role play (communication with a parent of a child with appendicitis)**	228	**4,27**	**0,61**
The role play was well-prepared.		4,45	0,79
The role play made it possible to practically apply the theoretical communication basics of interacting with patients.		4,32	0,78
There was enough time planned to conduct the role play.		4,11	1,15
The level of difficulty of the role play was appropriate.		4,21	0,93
The role play feedback discussion was helpful.		4,28	0,90
**Session 4 – Culturally determined beliefs and communication via interpreter**			
**Theoretical units**	164	**3,62**	**0,89**
Communication challenges when patients have language barriers		3,75	1,04
Types of interpreter and translation services		3,76	1,15
Difficulties caused by language barriers and resulting deficits in the treatment of patients with different cultural backgrounds		3,68	1,05
Skills and process tasks for achieving an effective communication via interpreter		3,27	1,13

Attachment 1 to: Gebhardt C, Mehnert-Theuerkauf A, Hartung T, Zimmermann A, Glaesmer H, Götze H. *COMSKIL: A communication skills training program for medical students*. GMS J Med Educ. 2021;38(4):Doc83. DOI: 10.3205/zma001479

**Role play (communication with elderly patients)[1]**	182	**4,14**	**0,70**
The role play was well-prepared.		4,22	0,93
The role play made it possible to practically apply the theoretical communication basics of interacting with patients.		4,15	0,89
There was enough time planned to conduct the role play.		4,37	1,00
The level of difficulty of the role play was appropriate.		3,90	0,99
The role play feedback discussion was helpful.		4,06	1,02
**Session 5 – Discussing prognosis and risk**			
**Theoretical units**	119	**3,69**	**0,64**
Challenges and difficulties of discussing prognostic information		4,05	0,69
Discussing different ways to convey prognostic information		3,41	0,92
Goals and strategies for providing prognostic information in an effective way		3,81	0,80
Skills and process tasks to convey prognostic information in an appropriate way		3,49	0,93
**Role play (patient with wound infection)**	113	**4,36**	**0,49**
The role play was well-prepared.		3,86	1,03
The role play made it possible to practically apply the theoretical communication basics of interacting with patients.		4,30	0,75
There was enough time planned to conduct the role play.		4,63	0,66
The level of difficulty of the role play was appropriate.		4,10	0,87
The role play feedback discussion was helpful.		4,40	0,84

Legend: 1= *strongly disagree*, 2= *somewhat disagree*, 3= *neither agree nor disagree* 4= *agree* 5= *strongly agree*)
[1] The role play „communication with elderly patients“ was postponed to the first part of the communication course. The role play „communication via interpreter“ was therefore added to session 4. The role play was recreated and therefore not part of the evaluation.
